# Two Dimensional-Difference in Gel Electrophoresis (2D-DIGE) Proteomic Approach for the Identification of Biomarkers in Endometrial Cancer Serum

**DOI:** 10.3390/cancers13143639

**Published:** 2021-07-20

**Authors:** Blendi Ura, Stefania Biffi, Lorenzo Monasta, Giorgio Arrigoni, Ilaria Battisti, Giovanni Di Lorenzo, Federico Romano, Michelangelo Aloisio, Fulvio Celsi, Riccardo Addobbati, Francesco Valle, Enrico Rampazzo, Marco Brucale, Andrea Ridolfi, Danilo Licastro, Giuseppe Ricci

**Affiliations:** 1 Institute for Maternal and Child Health, IRCCS Burlo Garofolo, 34137 Trieste, Italy; stefania.biffi@burlo.trieste.it (S.B.); lorenzo.monasta@burlo.trieste.it (L.M.); giovanni.dilorenzo@burlo.trieste.it (G.D.L.); federico.romano@burlo.trieste.it (F.R.); michelangelo.aloisio@burlo.trieste.it (M.A.); fulvio.celsi@burlo.trieste.it (F.C.); riccardo.addobbati@burlo.trieste.it (R.A.); giuseppe.ricci@burlo.trieste.it (G.R.); 2 Department of Biomedical Sciences, University of Padova, 35131 Padova, Italy; giorgio.arrigoni@unipd.it (G.A.); ilaria.battisti@studenti.unipd.it (I.B.); 3 Proteomics Center, University of Padova and Azienda Ospedaliera di Padova, 35131 Padova, Italy; 4 CRIBI Biotechnology Center, University of Padova, 35131 Padova, Italy; 5 Consorzio Sistemi a Grande Interfase, Department of Chemistry, University of Firenze, 50019 Firenze, Italy; francesco.valle@cnr.it (F.V.); marco.brucale@cnr.it (M.B.); andrea.ridolfi@ismn.cnr.it (A.R.); 6 Consiglio Nazionale delle Ricerche, Istituto per lo Studio dei Materiali Nanostrutturati (CNRISMN), 40129 Bologna, Italy; 7 Department of Chemistry “Giacomo Ciamician”, University of Bologna, 40126 Bologna, Italy; enrico.rampazzo@unibo.it; 8 Department of Chemistry, University of Firenze, 50019 Firenze, Italy; 9 ARGO Laboratorio Genomica ed Epigenomica, AREA Science Park, Basovizza, 34149 Trieste, Italy; danilo.licastro@cbm.fvg.it; 10 Department of Medical, Surgical and Health Sciences, University of Trieste, 34149 Trieste, Italy

**Keywords:** endometrial cancer, serum proteome, biomarkers, exosomes, 2D-DIGE

## Abstract

**Simple Summary:**

Endometrial cancer is the most common gynecologic malignancy arising from the endometrium. Identification of serum biomarkers could be beneficial for its early diagnosis. This study compared the serum proteins of 15 patients with endometrial cancer and 15 non-cancer subjects, identifying 16 proteins with diagnostic potential. Quantification of protein expression revealed the upregulation of CLU, ITIH4, SERPINC1, and C1RL in endometrial cancer samples compared to the sera of control subjects. We built a mathematical model based on this set of proteins that detects cancer samples with excellent sensitivity and specificity levels. After a validation phase our findings support the potential to use the proposed algorithm as a diagnostic tool in the clinical stage.

**Abstract:**

Endometrial cancer is the most common gynecologic malignancy arising from the endometrium. Identification of serum biomarkers could be beneficial for its early diagnosis. We have used 2D-Difference In Gel Electrophoresis (2D-DIGE) coupled with Mass Spectrometry (MS) procedures to investigate the serum proteome of 15 patients with endometrial cancer and 15 non-cancer subjects. We have identified 16 proteins with diagnostic potential, considering only spots with a fold change in %V ≥ 1.5 or ≤0.6 in intensity, which were statistically significant (*p* < 0.05). Western blotting data analysis confirmed the upregulation of CLU, ITIH4, SERPINC1, and C1RL in endometrial and exosome cancer sera compared to those of control subjects. The application of the logistic regression constructed based on the abundance of these four proteins separated the controls from the cancers with excellent levels of sensitivity and specificity. After a validation phase, our findings support the potential of using the proposed algorithm as a diagnostic tool in the clinical stage.

## 1. Introduction

Endometrial carcinoma (EC) is the most common malignancy of the female reproductive system and its incidence is increasing [1,2]. EC arises from the uterus lining and can be classified into two broad disease entities: endometrioid, affecting approximately 80% of patients; and non-endometrioid, afflicting about 20% of patients [3].

EC is a complex and heterogeneous disease at the epidemiological, clinical, pathological, and molecular levels [4]. To date, there are no tests for EC screening. Prompt diagnosis relies on rapid recognition of symptoms, with abnormal uterine bleeding (AUB) being the most common and specific, which is present in 90% of EC patients, and often forces patients to undergo unnecessary ultrasound and subsequent hysteroscopies [5]. A non-invasive test that can identify EC and reduce inappropriate and invasive examinations could transform patient care. In this regard, in a recent proof-of-concept study, O’Flynn and colleagues showed that EC could be detected in urine and vaginal fluids [6]. Prospective validation of these findings may support the embodiment of this non-invasive approach in clinical practice.

Molecular diagnostics are now essential in the management of cancer. The use of molecular biology techniques to analyze cancer patients’ biomarkers impacts the bedside for early detection, rational therapeutic targeting, and individual assessment of the risks and benefits of treatment. What is noteworthy in advanced and recurrent EC is that recent FDA approvals have highlighted the importance of treatment with new agents based on histologic features and biomarkers [7]. In this context, the use of EC serum proteomic analysis offers the potential to identify patients with actionable molecular alterations and further personalizes therapy. Among recent investigations, the results of a prospective single-site study of 100 patients with histologically confirmed endometrial cancer showed that EC serum HE4 level correlates with preoperative Magnetic resonance imaging MRI and intraoperative frozen section for identification of low-risk disease as established by the final histopathology [8]. Recently, several EC proteomic studies in serum identified potential biomarkers such as FAM83D [9], PAK1, and Rab 25 [10]. In addition to serum proteins, the serum exosome profiling significantly increased the number of biomarkers identified in the EC [11]. Exosomes are small 40–150 nm extracellular vesicles released by all cell types, including cancer cells [12]. These nanovesicles containing DNA, RNA, proteins and lipids are stable in biological fluids, including blood, urine, and saliva [13]. Amongst the examples, Song Y et al. identified LGALS3BP as a possible biomarker in the plasma exosome of EC [14]. In addition to serum studies, proteomic technologies explored the uterine aspirate to identify possible biomarkers [15,16]. However, despite significant advances in molecular profiling of EC-associated proteins, none has yet met the stringent requirements for clinical use.

The 2D-DIGE technology offers significant advantages in exploring a wide range of biological samples to identify target proteins that can act as a biomarker in cancer [17]. The 2D-DIGE investigated several types of tumors, including gastric cancer [18], liver cancer [19], and ovarian cancer [20], for the identification of dysregulated proteins. For instance, Pfetin was a successful case in identifying a tissue gastric cancer marker by using 2D-DIGE proteomics [21]. In the present study, we aimed to characterize the proteome of depleted serum in order to identify possible biomarkers in endometrial cancer by using 2D-DIGE coupled with MS.

## 2. Materials and Methods

### 2.1. Patients

A total of 30 patients (15 women suffering from EC and 15 non-EC controls) were recruited at the Institute for Maternal and Child Health—IRCCS “Burlo Garofolo” (Trieste, Italy)—during 2019 and 2020. All procedures complied with the Declaration of Helsinki and were approved by the Institute’s Technical and Scientific Committee. All patients signed informed consent forms. The clinical and pathological characteristics of the patients were described in Supplementary Table S1. The median age of patients was 45 years with a minimum of 36 and a maximum of 48 years. In the selection of controls, we excluded oncologic patients, Human immunodeficiency virus (HIV), Hepatitis B virus (HBV), Hepatitis C virus (HCV) seropositive subjects, and patients with leiomyomas or adenomyosis. In the selection of EC cases, we ruled out women with other oncologic pathologies, Human immunodeficiency virus (HIV), Hepatitis B virus (HBV), Hepatitis C virus (HCV) seropositive patients, and patients with leiomyomas or adenomyosis.

### 2.2. Serum Sample Collection and Enrichment

Blood was centrifuged at 5000 rcf × 5 min to obtain serum. After collection, serum was preserved at −80 °C. In order to improve the proteomic study performance, we used the ProteoMiner columns (Bio-Rad Laboratories, Inc., Hercules, CA, USA) to deplete the most abundant serum proteins. One ml of crude serum was incubated for 2 h at room temperature with Proteominer beads(Bio-Rad Laboratories, Inc., Hercules, CA, USA). After 3 cycles of washing with PBS, bound proteins were eluted from the column with TUC buffer: 7 M urea, 2 M thiourea, 4% CHAPS, and 50 mM Tris pH = 8.5. A second elution was performed by incubating the column with 4% SDS 100 mM beta-mercaptoethanol. The sample from the second elution was precipitated in methanol and chloroform. The pellets were dissolved in TUC buffer and reunited with the first fraction and the protein content was determined using the Bradford assay.

### 2.3. Sample Preparation for 2D-DIGE and Gel Image Analysis

After protein quantification, 50 µg of protein samples of depleted serum from endometrial cancer patients and controls were labelled with 400 pmol of either Cy5 or Cy3. The internal standard was prepared by pooling the sample and labelled with Cy2. The labelling reaction was carried out by incubating the samples on ice for 30 min in the dark. The reaction was stopped by adding 1 µL of 10 mM lysine. After labelling, the proteins were diluted in rehydration buffer: 7 M urea, 2 M thiourea, 2% (w/v) CHAPS, 65 mM DTT, and 0.24% Bio-Lyte (3–10)( Bio-Rad Laboratories, Inc., Hercules, CA, USA). The 2-DE analysis was conducted as previously described [22]. For 2-DE analysis, ReadyStrip™ 4–7 18 cm immobilized pH gradient (IPG)( Bio-Rad Laboratories, Inc., Hercules, CA, USA) strips were rehydrated at 50 V for 12 h at 20 °C and isoelectric focusing (IEF) was performed in a PROTEAN IEF Cell (Bio-Rad Laboratories, Inc., Hercules, CA, USA). For IPG strip equilibration, we performed two incubations: the first equilibration in a buffer (6 M urea, 2% SDS, 50 mM Tris-HCl (pH 8.8), 30% glycerol) for 5 min and a second equilibration step performed in 4% iodoacetamide for 10 min. After equilibration, the IPG strips were transferred to a 10% polyacrylamide gel (18.5 cm × 20 cm). After electrophoresis, 2-DE gels were scanned with a Molecular Imager PharosFX System(Bio-Rad Laboratories, Inc., Hercules, CA, USA). Molecular weights were determined by Precision Plus Protein Prestained Standards (Bio-Rad Laboratories, Inc., Hercules, CA, USA) covering a molecular weight range from 10 to 250 kDa. Two experimental replicates were performed. Gel analysis was completed using the MFA (multi fluorescence analysis) module of Proteomweaver 4.0 software (both from Bio-Rad Laboratories, Inc., Hercules, CA, USA) to normalize and quantify the protein spot. We used Ready strips 4–7 in pH instead of in the 3–10 pH range because most of the human proteins fall in this particular range of pI. Selecting a smaller pI range (but maintaining the strip length) allows the increase in 2-DE resolution and, as a consequence, the increase in the possibility to visualize lower abundance proteins [23].

### 2.4. Western Blotting

In order to confirm the 2D-DIGE results, we performed Western blotting in depleted serum and serum exosomes from the same patients, as previously described [24]. Additionally, the presence of some proteins identified as differentially abundant in the 2D-DIGE study (namely C1R, ITIH4, SERPINC1, and CLU) was also confirmed in tumor tissue: 4 tumor tissues were lysed in 1% NP-40, 50 mM Tris-HCl (pH 8.0), NaCl 150 mM with Phosphatase Inhibitor Cocktail Set II 1× (Millipore, Burlington, VT, USA) and 2 mM phenylmethylsulfonyl fluoride (PMSF), 1 mM benzamidine, and 30 µg of lysate were used for western blotting.

The proteins with fold change ≥ 2 were chosen for Western blotting validation. Briefly, 30 µg of proteins used for 2D-DIGE were loaded on 4–20% precast gel and then transferred to a nitrocellulose membrane. After protein transfer, the membrane was blocked by treatment with 5% defatted milk in TBS-tween 20 and incubated overnight at 4 °C with 1:600 diluted primary rabbit polyclonal antibody against ITIH4, with 1:300 diluted primary rabbit polyclonal antibody against C1R, with 1:300 diluted primary rabbit polyclonal antibody against SERPINC1, and with 1:500 primary mouse monoclonal antibody against CLU. For exosome markers, proteins were incubated with 1:300 primary mouse monoclonal antibody against CD9, with 1:1000 primary mouse monoclonal antibody against CD63, and with 1:1000 primary mouse monoclonal antibody against TSG101. All primary antibodies were purchased from Sigma-Aldrich (St. Louis, MO, USA).

After three washes were conducted with TBS-Tween 0.05% and membranes were incubated with HRP-conjugated anti-rabbit IgG and anti-mouse IgG (1:3000, Sigma-Aldrich; Merck KGaA, Darmstadt, Germany). The protein band signal was visualized using SuperSignal West Pico Chemiluminescent (Thermo Fisher Scientific Inc., Ottawa, ON, Canada). The intensities of the immunostained bands were normalized with the total protein intensities measured by staining the membranes from the same blot with Red Ponceau solution (Sigma-Aldrich, St. Louis, MO, USA).

### 2.5. Trypsin Digestion and MS Analysis

Preparative 2-DE gel (300 µg of loaded proteins) was run and stained with SYPRO Ruby and Coomassie colloidal Blue for protein visualization. Protein spots of interest from 2-DE were digested and analyzed by mass spectrometry as Ura et al. described [25]. Once excised from the gel, spots were washed four times with 50 mM NH_4_HCO_3_ and acetonitrile (ACN; Sigma-Aldrich, St. Louis, MO, USA) and dried under vacuum in a SpeedVac system. Three microliters of 12.5 ng/µL sequencing grade modified trypsin (Promega, Madison, WI, USA) in 50 mM NH_4_HCO_3_ were added for spot digestion and samples were digested overnight at 37 °C. Peptide extraction was conducted with three changes extraction by 50% ACN/0.1% formic acid (FA; Fluka, Ammerbuch, Germany). Peptide mixtures were dried under vacuum and stored at –20 °C until mass spectrometry (MS) analysis was performed. Samples were dissolved in 12 µL of 3% ACN/0.1% FA. Four microliters of each sample were investigated by LC-MS/MS with LTQ-Orbitrap XL mass spectrometer (Thermo Fisher Scientific, Waltham, MA, USA) coupled to a nano-HPLC Ultimate 3000 (Dionex—Thermo Fisher Scientific). Peptides were separated in a 10 cm pico-frit column (75 μm ID, 15 μm Tip; New Objective) packed in-house with C18 material (Aeris Peptide 3.6 µm XB-C18, Phenomenex). H_2_O/FA 0.1% and ACN/FA 0.1% were used as eluents A and B, respectively, and peptides were examined at a flow rate of 0.25 μL/min using a linear gradient of eluent B from 3% to 40% in 20 min. A Data Dependent Acquisition (DDA) was employed: A full scan between 300 and 1700 Da was performed at high resolution (60,000) on the Orbitrap. The ten most intense ions were then selected for CID fragmentation and MS/MS data acquisition in low resolution in the linear ion trap. Raw data files were analyzed with the software package Proteome Discoverer 1.4 (Thermo Fisher Scientific) and sought with Mascot Search Engine (version 2.2.4, Matrix Science, London, UK). The spectra were searched in the human section of the Uniprot database (July 2018 version) using the following parameters: enzyme specificity was set on trypsin with 1 missed cleavage allowed and the precursor and fragment ions tolerance were 10 ppm and 0.6 Da, respectively. Carbamidomethylcysteine and oxidation of methionine were formulated as a fixed modification and variable modification, respectively. The algorithm Percolator was used to assess the False Discovery Rate (FDR) at the protein and peptide level. We considered the hit proteins identified with at least three unique peptides with high confidence (FDR < 1%) as positive. In the case in which multiple proteins were identified within the same spot, the precursor area detection node of Proteome Discoverer was used to estimate the abundance of each protein.

### 2.6. Exosome Isolation

The isolation of the exosomes from crude serum was performed with the Total Exosome Isolation kit (Thermo Fisher Scientific, Waltham, MA, USA). The isolation was executed according to the manufacturer’s instruction. Briefly, 100 µL of crude serum was mixed with 20 µL reagent and incubated for 30 min at 40 °C. After incubation, samples were centrifuged at 10,000× *g* for 10 min at room temperature and resuspended at 25 µL of PBS and the protein content was determined using the Bradford assay.

### 2.7. Surface Preparation and Sample Deposition

The AFM images are used to estimate the size of the vesicles in the solution and characterized other biophysical properties. AFM is the only technique able to characterize this kind of sample at the nm level. The surfaces used for atomic force microscopy (AFM) imaging were poly-L-lysine (PLL)-coated borosilicate glass coverslips. The reagents were purchased from Sigma-Aldrich Inc (St. Louis, MO, USA)) unless otherwise stated. Microscopy glass slides (15 mm diameter round coverslips, Menzel Gläser) were cleaned in 3:1 H_2_SO_4_/H_2_O_2_ “Pirañha” solution and rinsed with ultrapure water. A further cleaning step consisted of sonicating the slides (Elmasonic Elma S30H sonicator bath) for 30 min in acetone, 30 min in isopropanol, and 30 min in ultrapure water (Millipore Simplicity UV). Before the experiments, the surfaces were exposed for 5 min to air plasma (Pelco Easiglow), then incubated for 30 min in a 0.1 mg/mL PLL solution in 100 mM borate buffer (pH 8.33) at room temperature and finally rinsed with ultrapure water and dried with nitrogen. The amount of 10 μL of the vesicle-containing solution was deposited on a PLL-functionalized glass slide and left to adsorb for 10 min at 4 °C and then inserted in the AFM fluid cell (see below) without further rinsing. The concentration of each sample was adjusted to maximize the surface density of the isolated individual vesicles and filaments.

### 2.8. Atomic Force Microscopy

AFM images were collected with a Multimode8 microscope (Bruker, USA) equipped with a Nanoscope V controller and JV piezo scanner. Samples were measured in a sealed fluid cell filled with ultrapure water or PBS using ScanAsyst Fluid + probes (Bruker, USA).

### 2.9. Image Analysis

The raw images were processed with Gwyddion 2.59 [26] for background subtraction. Quantitative morphometry of EVs was performed as described elsewhere [27]. Concerning the fibers, the height and pitch of their helicity were respectably measured by Height Distribution and by the Image Profile tool.

### 2.10. Bioinformatic Analysis

Proteins, identified by MS, were analyzed by PANTHER classification systems. Proteins were then categorized according to their involvement in the biological processes, molecular function, and protein class. For pathways classification, the REACTOME tool was adopted. The Ingenuity Pathway Analysis (IPA) was employed to generate bio-functions [28]. In IPA, we considered *p* < 0.01 as a statistically significant value. For the filter summary, we only considered associations where confidence was high (predicted) or that had been observed experimentally.

### 2.11. Statistical Analysis

Differences were considered significant when spots showed a fold change ± 1.5 and satisfied the Mann–Whitney sum-rank test (*p* < 0.05). In order to better assess if the expression of specific proteins was associated with EC, a multivariate logistic regression analysis was conducted to determine if proteins found to be upregulated or downregulated in this study could be used as predictive markers for the tumor. All analyses were managed with Stata/IC 16.1 for Windows (StataCorp LP, College Station, TX, USA).

## 3. Results

### 3.1. Proteomic Study

**Figure 1:**
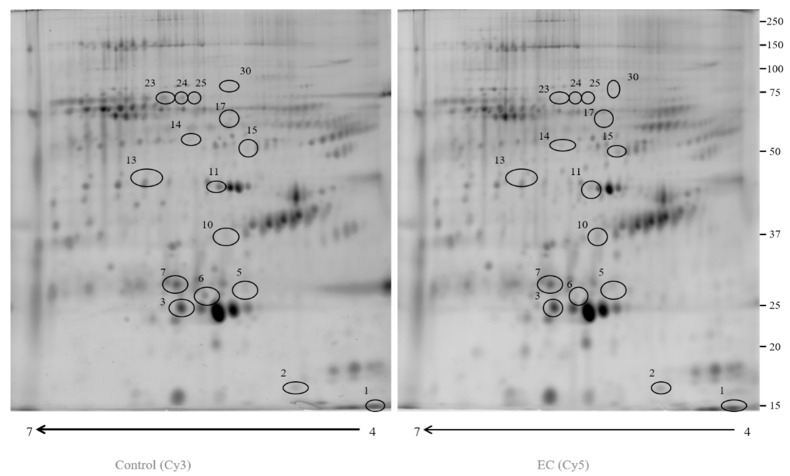
A 2D-DIGE map of depleted serum from control serum and endometrial serum. IPG strips 4–7 were used for the first dimension and 10% SDS-PAGE was utilized for the second dimension. The numbered circles indicate the differently abounding spots.

In this study, we used 2D-DIGE coupled with MS to compare the proteomic profile of depleted serum of 15 ECs (Cy5) and 15 controls (Cy3). More than 2400 protein spots (Figure 1) were detected in both types of samples. After imaging and statistical analysis, 16 protein spots showed a significant alteration (*p* < 0.05) of their volume in EC vs. control samples, with a fold change of ≥1.5 or ≤0.6 (Table 1). The spots were subjected to in-gel digestion, LC-MS/MS analysis, and proteins were identified by searching the MS/MS data in the human section of the UniProt database. Six proteins showed a fold change ≥ 1.5 (CLU, SERPINC1, ITIH4, C1R, APOC3, and DSC1) and ten showed a fold change ≤ 0.6 (APCS, C9, APOA1, ALB, ITIH2, APOA4, ITIH2, CFHR1, ITIH2, and ACTB). All parameters functional to assess the quality of peptide and protein identifications were reported as supplementary data (File S1).

### 3.2. Western Blotting for Data Validation

**Figure 2:**
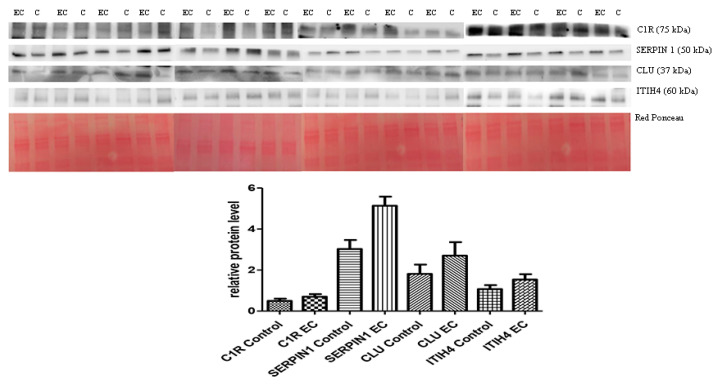
Western blotting analysis of four proteins ITIH4, CLU, SERPIN1, and C1R in controls (C) and endometrial cancer (EC) patients. The intensity of immunostained bands was normalized against the total protein intensities measured from the same blot stained with Red Ponceau (Densitometry Readings available as Supplementary File S2). The graph shows the relative abundance of proteins in control and endometrial cancer serum. Only SERPIN1 levels were significantly associated with EC (*p* = 0.010). Results are shown as a histogram (*p* < 0.05) and each bar represents mean ± standard deviation (C1R control mean = 0.49 ± 0.42, C1R ADK mean = 0.7 ± 0.48; SERPIN1 control mean = 3.02 ± 1.7, SERPIN1 ADK mean = 5.1 ± 0.71; CLU control mean = 1.81 ± 1.77, CLU ADK mean = 2.7 ± 2.55; ITIH4 control mean = 1.07 ± 0.74, ITIH4 ADK mean = 1.53 ± 1.02).

The 2D-DIGE experiments on depleted serum were validated by Western blotting and proteins that showed a fold change ≥ 2 by DIGE were selected, namely ITIH4, CLU, C1R, and SERPINC1. The abundance of these proteins in the depleted serum of 15 controls was compared with 15 from ECs. Quantitative analysis was consistent with 2D-DIGE, showing a higher serum abundance in EC patients than in controls (see Figure 2) (an uncropped version of this image on Supplementary File S3).

The higher abundance was notable for SERPIN1 (*p*= 0.0015) but was not statistically meaningful for the other proteins: ITIH4 (*p* = 0.1897), C1R (*p* = 0.0624), and CLU (*p* = 0.1228). We then conducted a bivariate logistic regression analysis to study the association between EC and the putative markers. Again, only SERPIN1 was significantly associated (OR2.02; 95% CI 1.19–3.45, *p* = 0.010). Subsequently, we included all the markers in a multivariate logistic regression and applied a step-down procedure and eliminated the markers with *p* ≥ 0.05. The C1R marker had a *p* = 0.991 and was therefore excluded. All other markers (CLU, ITIH4, and SERPIN1) were significantly associated with the outcome and remained so after the exclusion of C1R (Table 2).

We built a predictive model showing that these markers could be employed to obtain a prediction for EC with 100% sensitivity and 86.67% specificity with AUC = 0.9289. The model possessed the following formula.

Predict_EC = 1/(1 + exp(−(−11.63145 + 2.842529∗ITIH4 + 1.321026∗SERPINC1 + 0.9175675∗CLU)))(1)

According to the present model and data, a value ≥ 0.4835539 could determine with 100% sensitivity and 86% specificity that the subject had EC.

**Figure 3:**
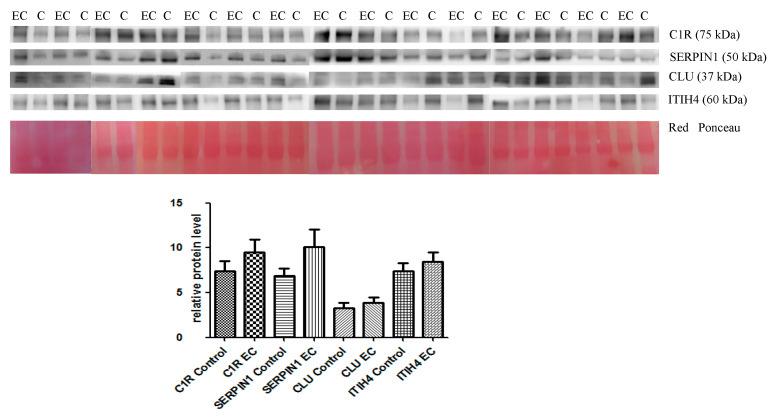
Western blotting analysis of the four proteins ITIH4, CLU, SERPIN1, and C1R in control (C) and endometrial cancer (EC) serum exosome. The intensity of immunostained bands was normalized against the total protein intensities measured from the same blot stained with Red Ponceau (Densitometry Readings available as Supplementary File S2). The graph shows the relative abundance of proteins in control and endometrial cancer serum exosomes. The levels of the proteins were not significantly associated with EC. Results are shown as a histogram, each bar representing mean ± standard deviation (C1R control mean = 7.42 ± 4.25, C1R ADK mean = 9.51 ± 5.33; SERPIN1 control mean = 6.84 ± 3.41, SERPIN1 ADK mean = 10.09 ± 7.57; CLU control mean = 3.24 ± 2.47, CLU ADK mean = 3.87 ± 2.39; ITIH4 control mean = 0.27 ± 0.23, ITIH4 ADK mean= 0.39 ± 0.34).

The abundance of the same proteins was validated in the serum exosome of the same patients. Western blotting confirmed the 2D-DIGE results, revealing a higher serum abundance in EC patients than in controls (see Figure 3) (an uncropped version of this image on Supplementary File S3).

The increase was not statistically significant. Subsequently, in order to determine whether the levels of these proteins in the exosome could be used as a marker for EC, a multivariate logistic regression analysis was carried out. The analysis proved that two markers (SERPINC1 and CLU) were not significantly associated with EC. Applying a step-down procedure, SERPINC1 was first dropped and the analysis repeated and again CLU was not significantly associated with EC and was subsequently abandoned. With the two remaining markers (C1RL and ITIH4), we generated a predictive model, which, unlike the previous model on serum proteins, could only achieve 80% sensibility and 86.67% specificity (AUC = 0.8089) (Table 3).

In an analogous case with the previous analysis and even in this case, it would be possible to adopt this model to predict the likeliness of EC by using the coefficients from the multivariate logistic regression in the following equation.

Predict_EC = 1/(1 + exp(−(−4.359769 + 5.040988∗exo_ITIH4 + 0.31399∗exo_C1RL)))(2)

If the value obtained is over ≥ 0.56, it would then be possible to determine with 80% sensitivity and 86,67% specificity that the subject suffers from EC.

**Figure 4:**
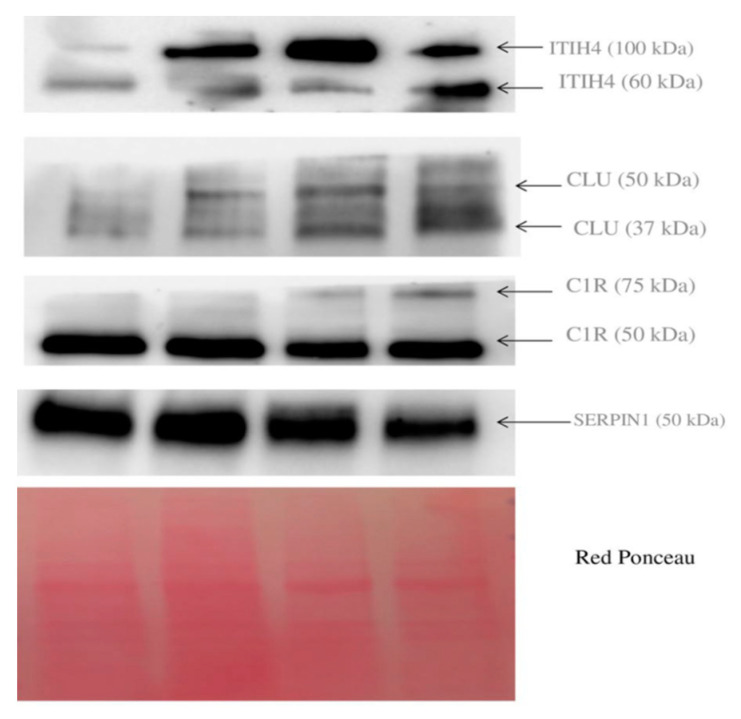
Western blotting of protein ITIH4, CLU, C1R, and SERPIN1 present in tumor tissue.

We performed Western blotting to validate the presence of these proteins in four tumor tissues. As shown in Figure 4, the protein ITIH4 was found in two proteoforms at 100 and 60 kDa. CLU was found in two proteoforms at 50 and 37 kDa. C1R was found again as a double band at 75 and 50 kDa, while SERPINC1 was detected as a single band at 50 kDa. The MW of these proteins in tissue correspond to the apparent MW that was found in our 2D-DIGE maps of serum, thus confirming that these various proteoforms are probably leaking from tumor cells and are not the result of a degradative process taking place in blood.

### 3.3. Atomic Force Microscopy (AFM) Imaging

**Figure 5:**
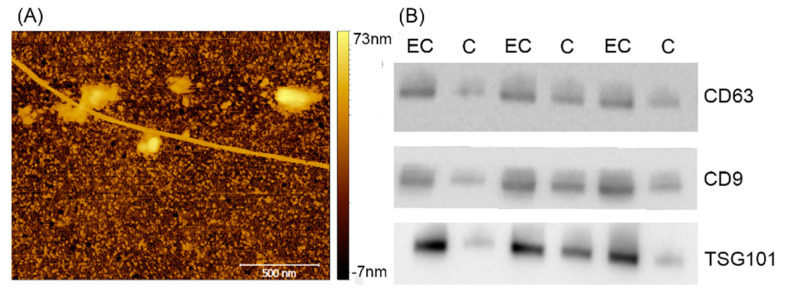
(**A**) Atomic Force Microscopy (AFM) imaging of endometrial serum exosome. (**B**) CD 63, CD9, and TSG101 common exosome marker. Western blotting in exosome of three C (control) and three EC (endometrial cancers).

The images collected on this sample displayed extracellular vesicles (EVs) in clusters in contact with long filaments (Figure 5). The absence of individual and isolated EVs hindered the possibility of performing a single vesicle analysis, as shown in previous works [26]. Therefore, we measured only its height in the typical exosome range of 60–100 nm. On the other hand, filaments were always a few µm long and (9.1 ± 2) nm high and exhibited a left-wing helicity of (290 ± 40) nm.

### 3.4. Bioinformatic Analysis

**Figure 6:**
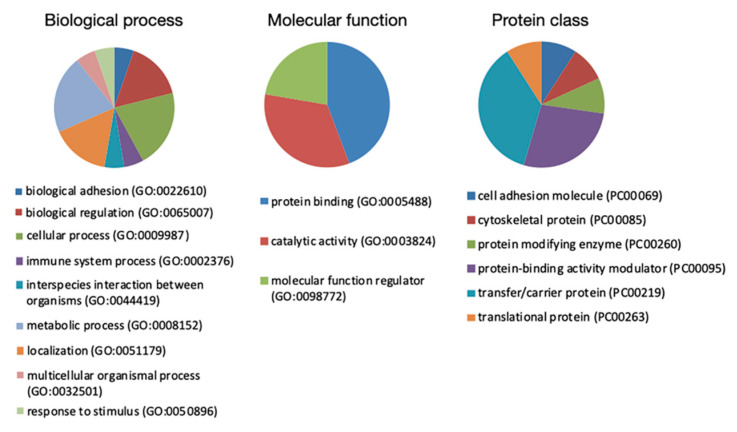
PANTHER classification of identified proteins in the EC serum according to their biological process, molecular function, and protein class.

Proteins of interest identified by MS were subjected to Protein Analysis Through Evolutionary Relationships (PANTHER) classification, which categorized them into groups according to their biological processes, molecular function, and protein class (Figure 6). According to their biological processes, proteins were grouped into four main categories: biological regulation, cellular process, localization, and metabolic process. For molecular function, proteins were classified into protein binding, catalytic activity, and molecular function regulator. For protein class, proteins were categorized into transfer/carrier protein, protein binding activity modulator, and protein modifying enzyme.

**Figure 7:**
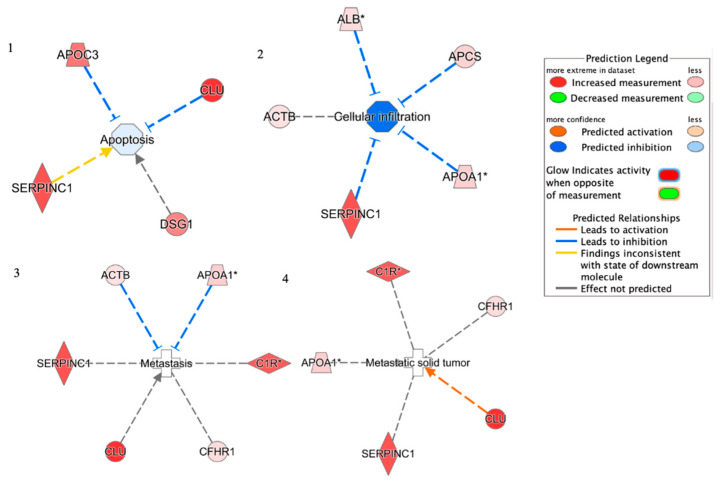
Network build-up from one of the most significant bio-functions: (**1**) Apoptosis; (**2**) Cellular infiltration; (**3**) Metastasis; (**4**) Metastatic solid tumor.

By using REACTOME, proteins were grouped into five main pathways: Plasma lipoprotein remodeling (APOA4, APOA1, APOC3, and ALB), Complement cascade (C1R, CFHR1, C9, APCS, and CLU), Post-translational protein phosphorylation (APOA1, ITIH2, SERPINC1, and ALB), Regulation of Insulin-like Growth Factor (IGF) transport (APOA1, ITIH2, SERPINC1, and ALB). Proteins were used in the core analysis with IPA software. The top networks in which these proteins were required corresponded to (1) Apoptosis; (2) Cellular infiltration; (3) Metastasis; (4) Metastatic solid tumor (Figure 7). Four proteins were implicated in the Apoptosis network: SERPINC1, APOC3, DSG1, and CLU. Five were included in Cellular infiltration: SERPINC1, ALB, ACTB, APOA1, and APCs. Six were involved in the Metastasis network: SERPINC1, ACTB, APOA1, C1R, CLU, and CFHR1. Five were related to the Metastatic solid tumor: SERPINC1, CLU, CFHR1, C1R, and APOA1.

## 4. Discussion

Despite significant advances in discovering biomarkers for EC, no one has reached clinical validation so far. Diagnostic biomarker validation is a crucial challenge in biomarker discovery, mainly related to the lack of assay generalizability or bias [29]. Since sources of bias are related to the study design and technology used, it is fundamental to clearly define eligibility criteria for patient selection, apply standard operating procedures to samples, and to ensure sufficient statistical power to determine diagnostic accuracy. The critical aspects of the analytical method employed for the validation could be improved by applying various technologies to biomarker assay development. So far, a few studies on serum endometrial cancer have validated biomarkers with immunochemical methods [9,30].

To the best of our knowledge, this is the first study coupling ProteoMiner, 2D-DIGE, and MS for the identification of biomarkers in endometrial cancer. Among the 16 potential biomarkers identified in depleted serum, we validated four proteins, namely ITIH4, CLU, SERPIN1, and C1R, in all the collected samples. Although the trend in abundance of the validated proteins resulting from Western blotting was consistent with that found by 2D-DIGE, only SERPIN1 was statistically significant when taken alone. This occurrence could be due to the difference between the two methodologies and the comparison of two data sources. Of note, a predictive set of the four proteins (ITIH4, CLU, SERPIN1, and C1R) allowed us to separate cases from controls using multivariate logistic regression analysis, with an AUC of 0.9289. The validation of the same four proteins in the serum exosome provided consistent results in the abundance’s trend, suggesting that the two sources (depleted serum and exosomes) maintained the abundance trend of these proteins. The predictive logistic regression of the same proteins in the exosomes showed an AUC of 0.8089 with only two markers retained by the model.

By comparing our data with the ones reported by Tarney and colleagues, who conducted a proteomic study on EC serum by using Tandem Mass Tag (TMT) platform, we could confirm that APOA1, APOA4, and CFHR1 were downregulated in the EC serum [30]. We also performed our Reactome pathway analysis, revealing the dysregulation of several pathways. In particular, due to the vital role of the complement cascade in influencing tumor growth and spread [30], the enrichment of the proteins set in this pathway probably represented a plausible mechanism that considered the progression of the disease. Specifically, CLU was an extracellular chaperone that prevented aggregation of non-native proteins while promoting metastasis in solid tumors and retaining an anti-apoptotic capacity [28,30]. These data complied with our IPA analysis.

APCS played a significant role in the phagocytic clearance of apoptotic cells. It was overexpressed in cancer cells upon treatment with photodynamic therapy (PDT) and allowed the clearance of the dead cells induced by the treatment [31,32]. C1R was another protein identified in our study as a component of the Complement cascade. It was a serine protease that combined with C1q and C1s to form C1, which is the first component of the complement system [33]. This enzyme promoted the growth of solid tumors such as cutaneous squamous cell carcinoma [34].

An increasing body of evidence suggested that metabolic dysregulation increased the risk of endometrial cancer [35]. Our findings, by pointing to a variation in the abundance of several proteins involved in the plasma lipoprotein remodeling pathway, strengthens the notion of a critical role of metabolism in cancer development [36]. ApoA4 was synthesized by the small intestine and secreted into intestinal lymph during fat absorption [37]. This protein had an anti-inflammatory activity protecting from cardiovascular diseases. Moreover, it had a crucial role in modulating glucose metabolism resulting in a possible correlation between protein and tumor development [38,39]. ApoA1 was the major component of HDL particles in plasma and participated in the transport of cholesterol from tissues to the liver for excretion [40]. Considering that oxidative stress [41] and inflammation [42] were strongly associated with cancer growth, the ApoA1 antioxidant and anti-inflammatory activities were of interest in the context of cancer progression [43].

The Reactome analysis also identified several key proteins known to be involved in the Regulation of Insulin-like Growth Factor (IGF) transport that are essential for growth, angiogenesis, and metastatic activities in various cancers [44,45,46]. For instance, SERPINC1 was a serine protease inhibitor in plasma that regulated the blood coagulation cascade [47], and exhibited anti-apoptotic, pro-angiogenic, and inflammatory activities [48]. Our results of the IPA analysis, consistent with data reported in the literature, indicates pro-metastatic activity in solid tumors.

We believe our current data can help improve various aspects of the diagnostic process. In a subsequent study, it will be possible to examine whether the proposed algorithm can be employed in the prognostic staging of EC. For instance, it would be interesting to investigate these biomarkers in the likelihood of hyperplasia regression or cancer early stages [49]. Furthermore, the development of predictive biomarkers has gained considerable interest in advancing the application of precision medicine in gynecological oncology and overcoming the issues related to the chemotherapy response and relapse risk.

## 5. Conclusions

We believe that our current data might expand the knowledge for the discovery of biomarkers in EC serum. We identified a set of putative biomarkers in serum and identified an algorithm that can separate controls from cancer patients with high sensitivity and specificity. Moreover, our current findings suggest an association between the trend of protein abundance in depleted serum and in serum exosomes. These features can pave the way for the creation of a diagnostic test.

## Figures and Tables

**Table 1:** Different abundance proteins identified by mass spectrometry in EC compared to the control serum.

Accession Number	Spot Number	Protein Description	Gene Symbol	Protein Score	Fold Change *	*p*-Value
P10909	10	Clusterin	CLU	353.36	2.25	0.014
P01008	15	Antithrombin	SERPINC1	1165.24	2.2	0.024
Q14624	14	Inter-alpha-trypsin inhibitor heavy chain H4	ITIH4	566.84	2.1	0.007
A0A3B3ISR2	30	Complement subcomponent C1r	C1R	366.02	2	0.036
P02656	1	Apolipoprotein C-III	APOC3	971.08	1.7	0.034
Q02413	2	Desmoglein-1	DSG1	340.05	1.6	0.017
P02743	7	Serum amyloid P-component	APCS	1525.76	0.6	0.02
P02748	17	Complement component C9	C9	712.30	0.59	0.04
P02647	3	Apolipoprotein A-I	APOA1	672.27	0.5	0.027
A0A0C4DGB6	5	Albumin	ALB	191.37	0.48	0.007
Q5T985	24	Inter-alpha-trypsin inhibitor heavy chain H2	ITIH2	620.93	0.44	0.04
P06727	6	Apolipoprotein A-IV	APOA4	566.64	0.4	0.01
Q5T985	25	Inter-alpha-trypsin inhibitor heavy chain H2	ITIH2	691.01	0.38	0.005
B1AKG0	13	Complement factor H-related protein 1	CFHR1	252.88	0.38	0.012
Q5T985	23	Inter-alpha-trypsin inhibitor heavy chain H2	ITIH2	792.30	0.32	0.04
P60709	11	Actin, cytoplasmic 1	ACTB	447.69	0.3	0.01

* Fold change was defined as the mean % volume ratio according to the formula: %V = Volume single spot/Volume total spot of EC vs. C.

**Table 2:** Multivariate logistic regression model for EC biomarkers using serum protein levels and resulting after the step-down procedure; only significantly associated variables are kept (*p* < 0.05).

Marker	Odds Ratio	OR 95% CI	*p*-Value	Regression Coefficients	RC 95% CI
ITIH4	17.16	1.51–194.19	0.022	2.84	0.41–5.27
SERPINC1	3.74	1.46–9.59	0.006	1.32	0.37–2.26
CLU	2.50	1.16–5.39	0.019	0.91	0.18–1.68

Note: Refined model (excluding C1R). OR 95% CI: 95% confidence intervals of the Odds Ratio. RC 95% CI: 95% confidence intervals of the regression coefficients.

**Table 3:** Multivariate logistic regression model for EC biomarkers using exosome protein levels and resulting after step-down procedure. Only significantly associated variables are kept (*p* < 0.05).

Marker	Odds Ratio	OR 95% CI	*p*-Value	Regression Coefficients	RC 95% Cl
ITIH4	154.62	2.01–11,866.05	0.023	5.04	0.69–9.38
C1R	1.37	1.04–1.79	0.024	0.31	0.04–0.58
Constant	0.013	0.0003–0.486	0.019	−4.34	−8.11–−0.72

Note: Refined model (excluding CLU and SERPINC1). OR 95% CI: 95% confidence intervals of the Odds Ratio. RC 95% CI: 95% confidence intervals of the regression coefficients.

## Data Availability

The data presented in this study are available on request from the corresponding author. The data are not publicly available due to ethical reasons.

## Supplementary Materials

The following are available online at https://www.mdpi.com/article/10.3390/cancers13143639/s1, File S1: Supplementary proteomic data, File S2: Densitometry Reading intensity ratio, File S3: uncropped images of western blot presented in Figure 2 and Figure 3, Table S1: Clinico-pathological characteristics of the 30 women enrolled in the study. The patients enrolled are all endometroid cancer type 1.
