# Supplementary material for: Two Dimensional-Difference in Gel Electrophoresis (2D-DIGE) Proteomic Approach for the Identification of Biomarkers in Endometrial Cancer Serum
Source: Cancers (Basel). 2021 Jul 20;13(14):3639. doi: 10.3390/cancers13143639 (PMC8305989; doi:10.3390/cancers13143639)
Supplement: Supplementary file 1 [file cancers-13-03639-s001.zip › Supplementary file S3.pdf]

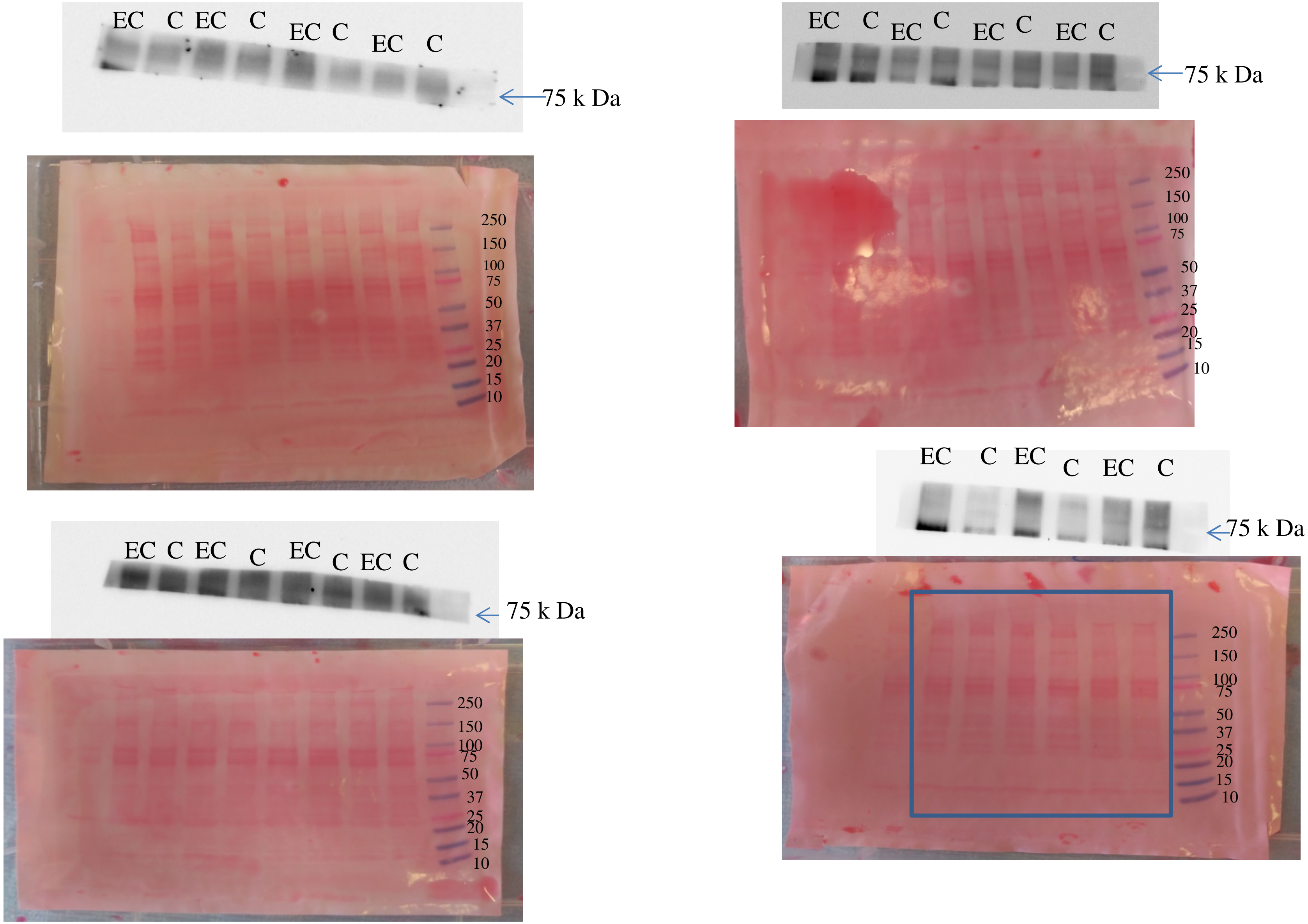

Whole membrane of C1R protein from depeted serum

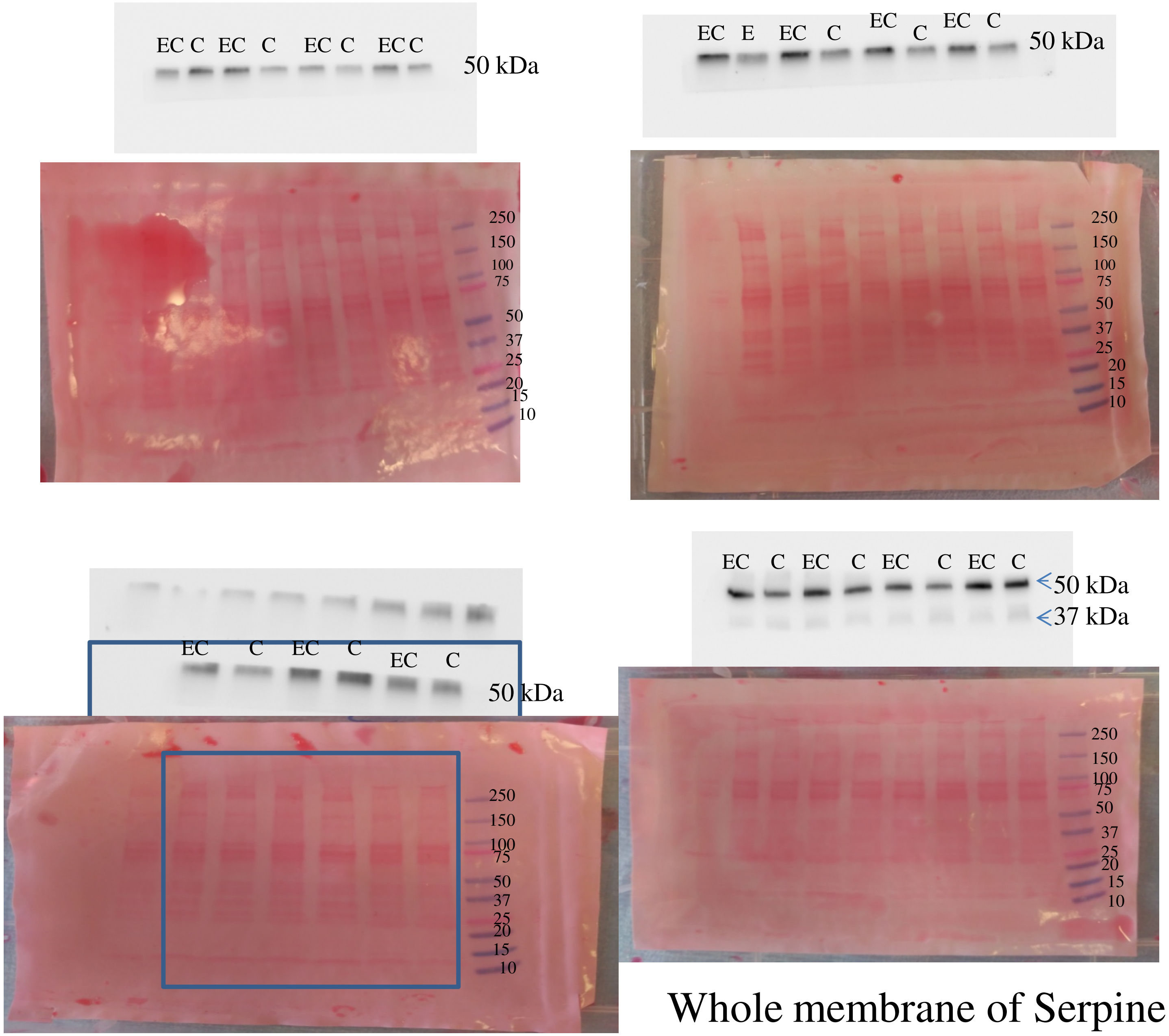

Whole membrane of Serpine1protein from depleted serum

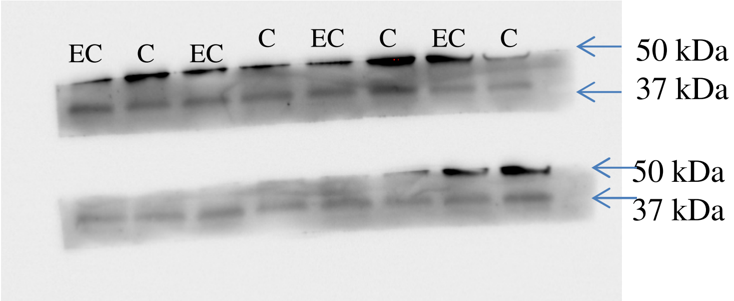

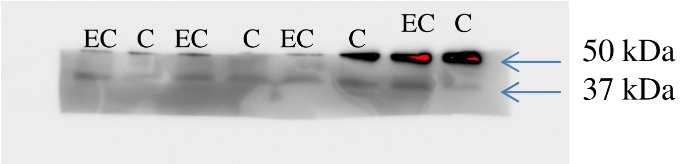

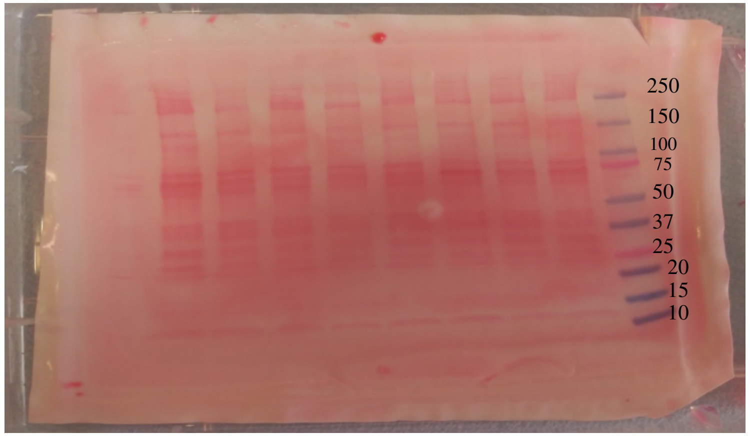

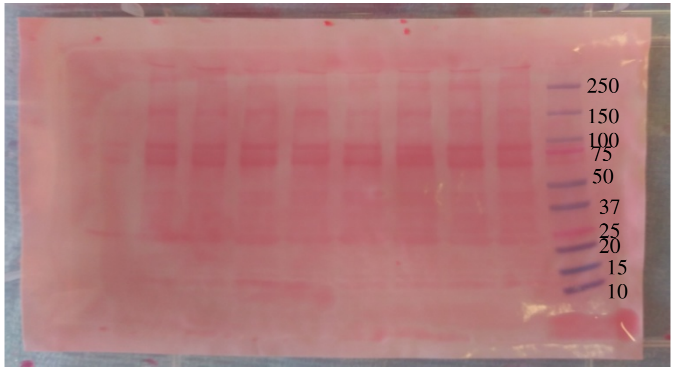

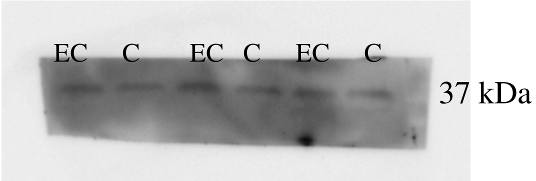

Whole membrane of CLU protein from depeted serum

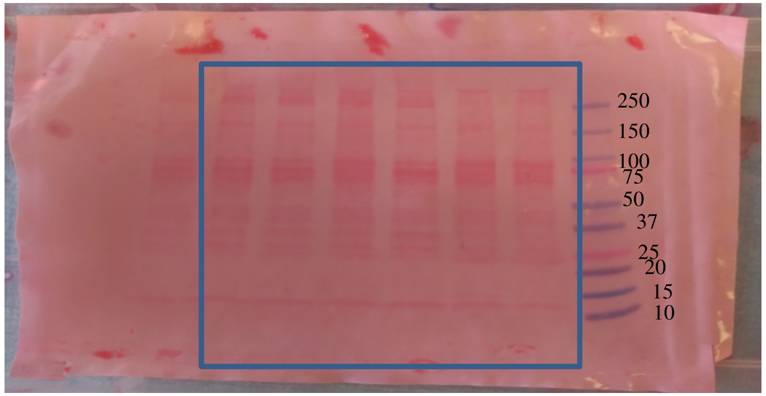

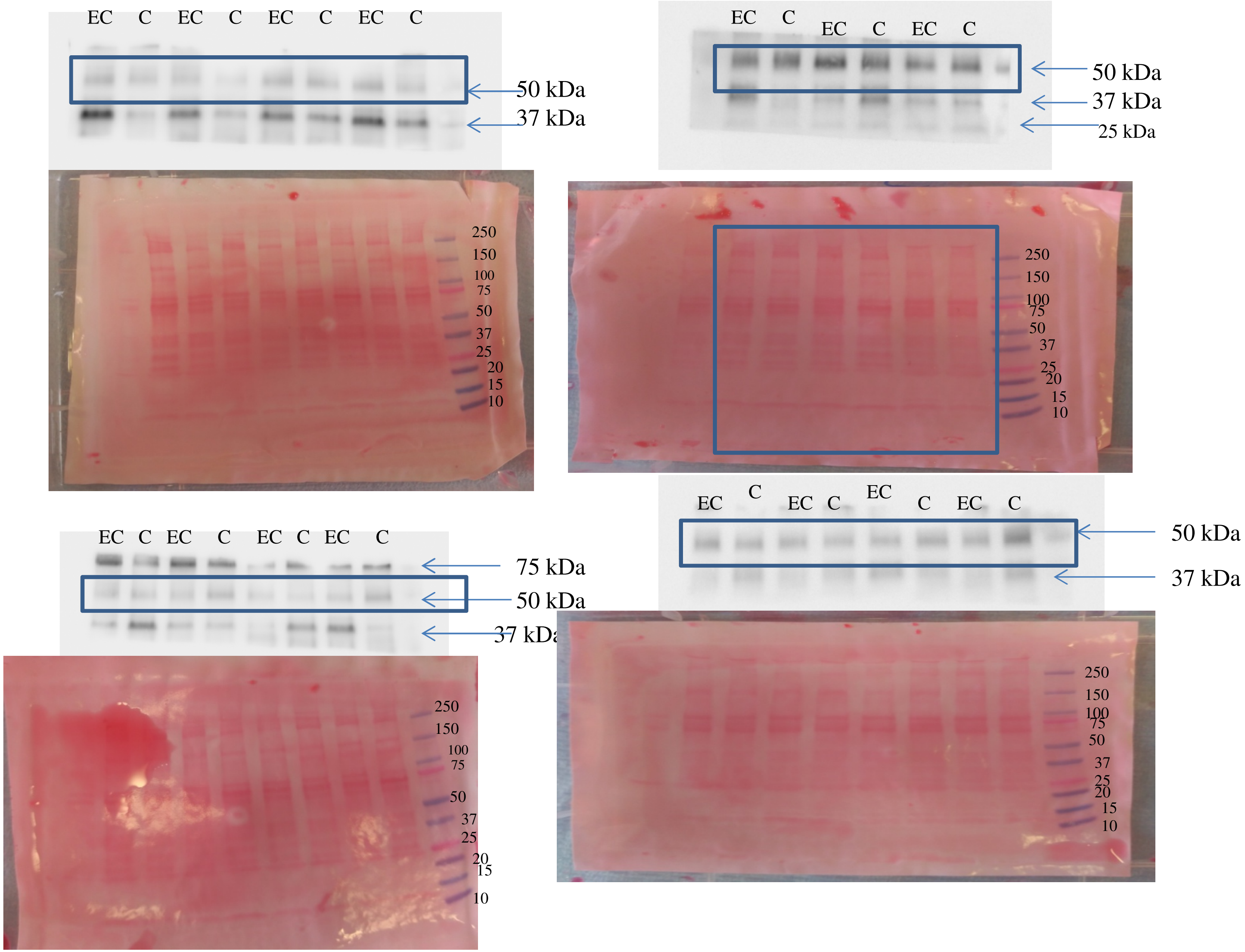

Whole membrane of ITIH4 protein from depleted serum

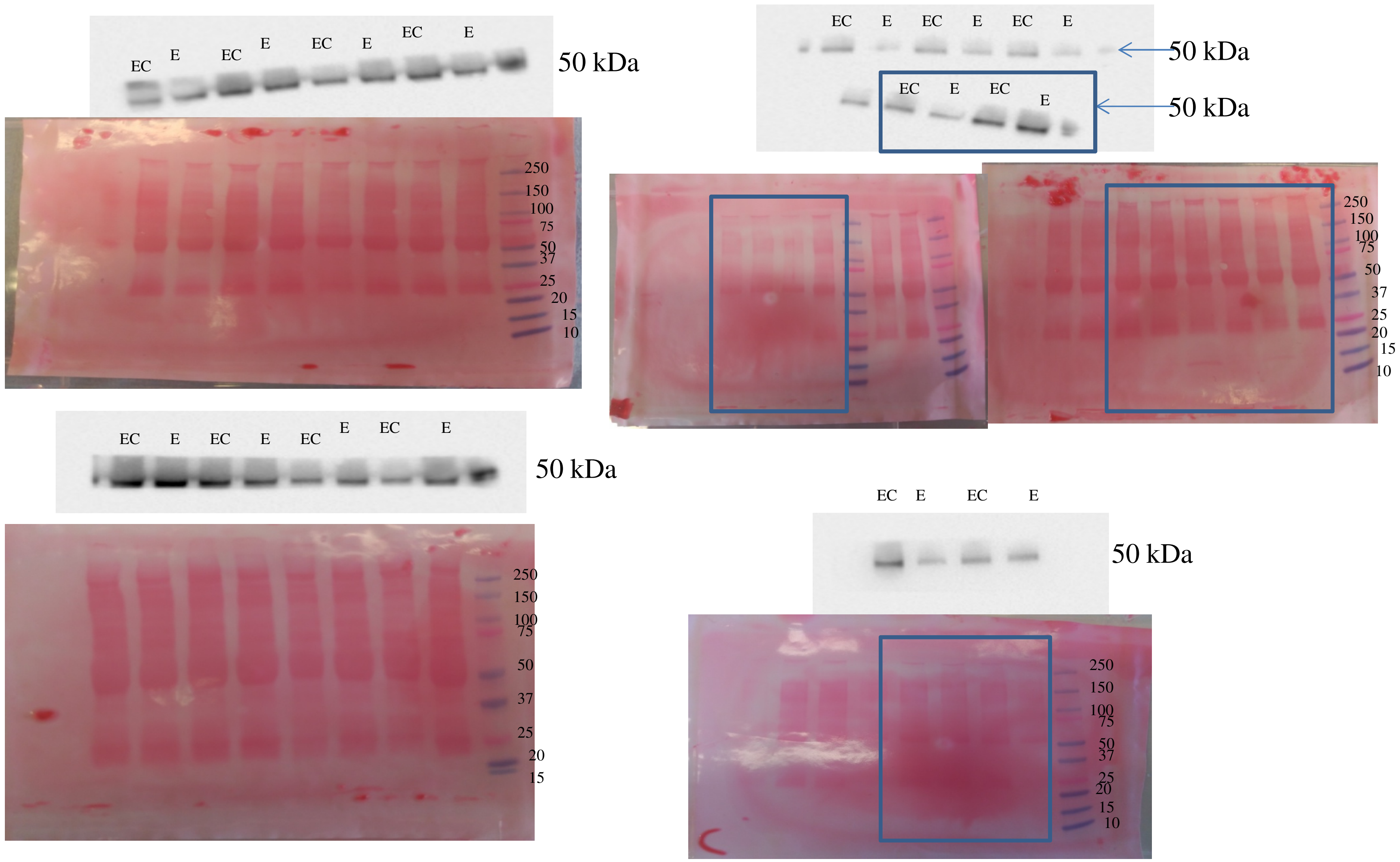

Whole membrane of Serpine1 protein from exosome

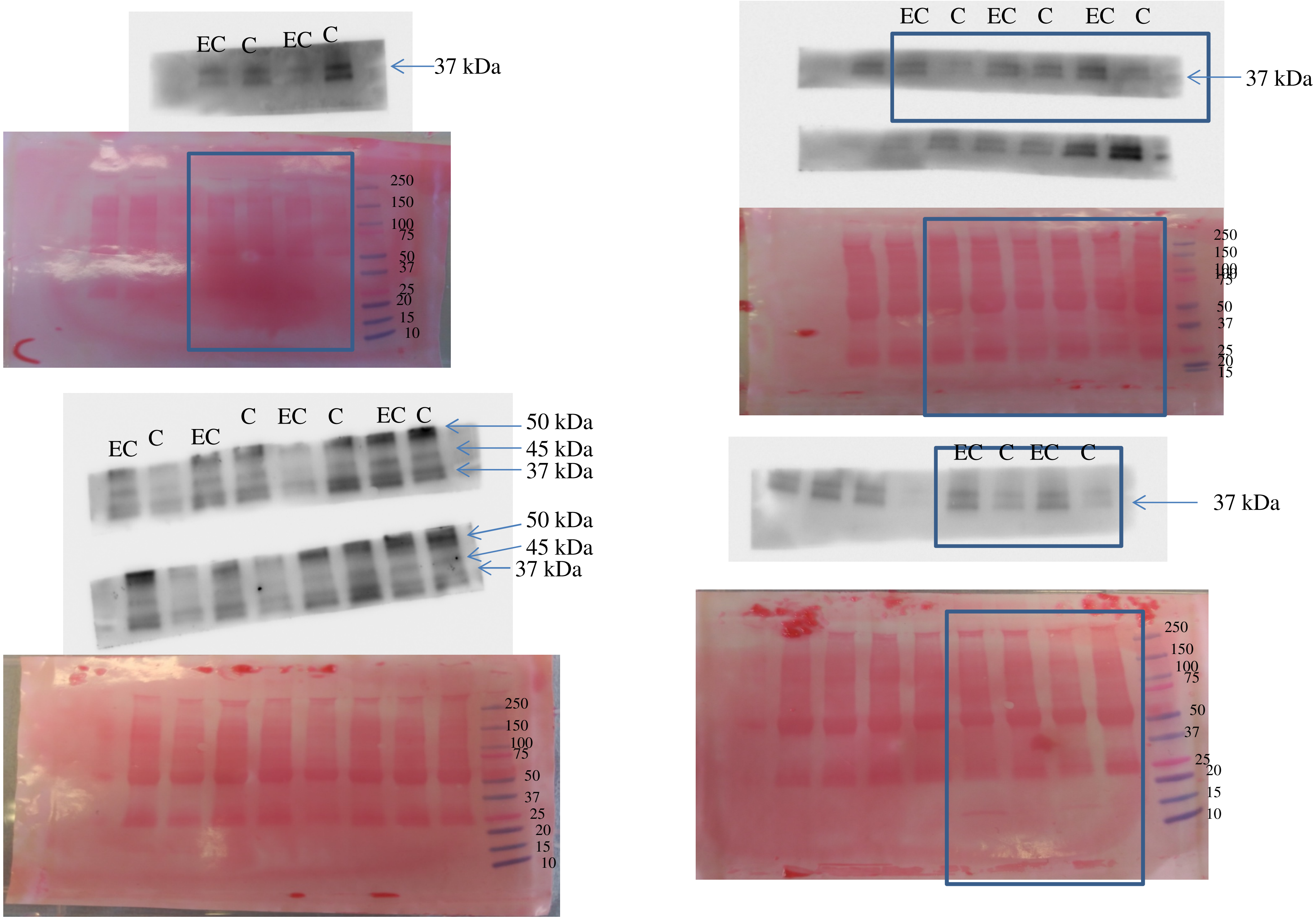

Whole membrane of CLU protein from exosome

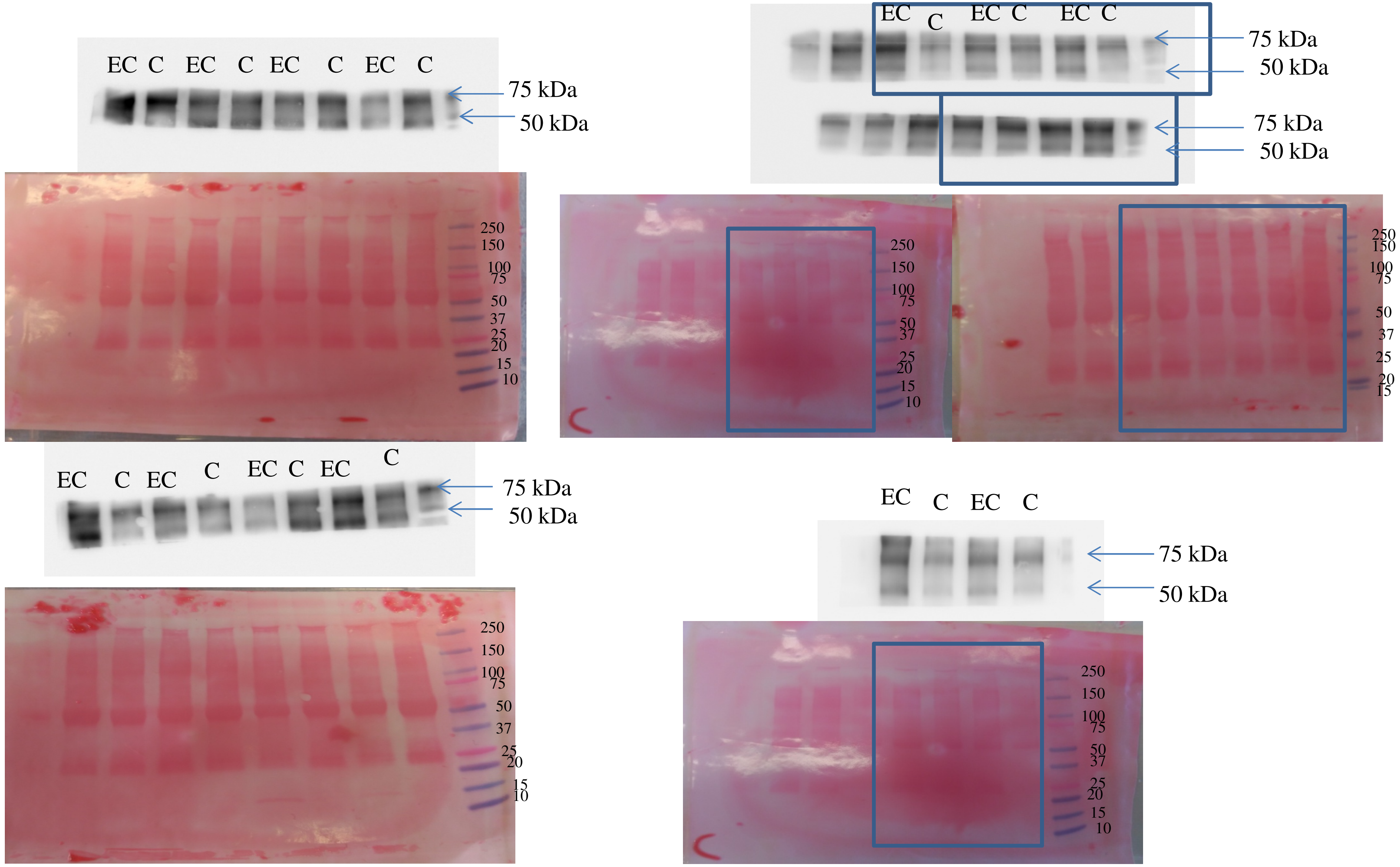

Whole membrane of C1R protein from exosome

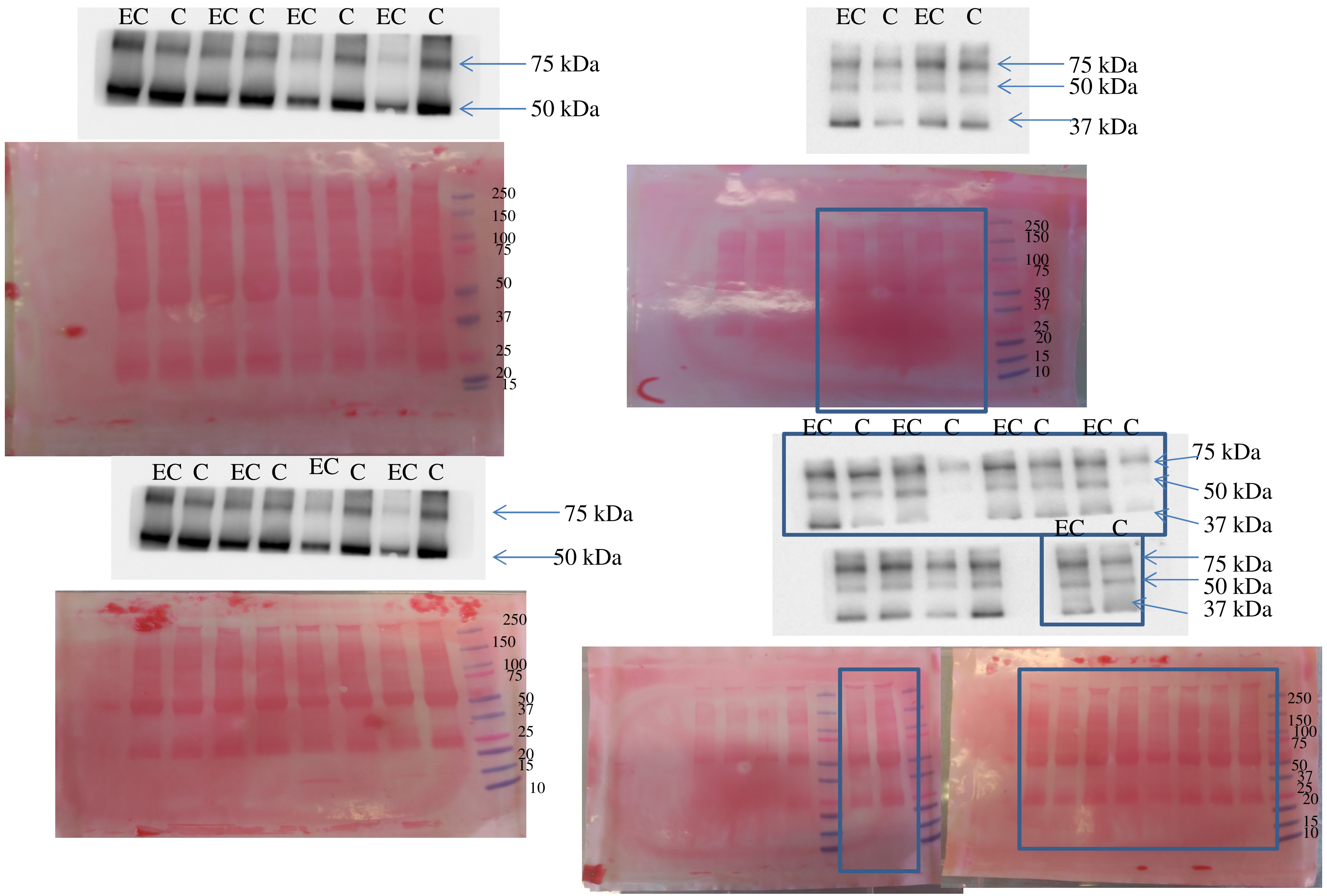

Whole membrane of ITIH4 protein from exosome
